# Differential regulation of mTORC1 and mTORC2 is critical for 8-Br-cAMP-induced decidualization

**DOI:** 10.1038/s12276-018-0165-3

**Published:** 2018-10-30

**Authors:** Mi-Ock Baek, Hae-In Song, Joong-Soo Han, Mee-Sup Yoon

**Affiliations:** 1 Department of Molecular Medicine, School of Medicine, Gachon University, Incheon, 21999 Republic of Korea; 2 Biomedical Research Institute and Department of Biochemistry and Molecular Biology, College of Medicine, Hanyang University, Seoul, 04763 Republic of Korea

**Keywords:** Differentiation, Growth factor signalling, Infertility

## Abstract

Human endometrium decidualization, a differentiation process involving biochemical and morphological changes, is a prerequisite for embryo implantation and successful pregnancy. Here, we show that the mammalian target of rapamycin (mTOR) is a crucial regulator of 8-bromoadenosine 3’,5’-cyclic monophosphate (8-Br-cAMP)-induced decidualization in human endometrial stromal cells. The level of mSin1 in mTOR complex 2 (mTORC2) and DEPTOR in mTOR complex 1 (mTORC1) decreases during 8-Br-cAMP-induced decidualization, resulting in decreased mTORC2 activity and increased mTORC1 activity. Notably, DEPTOR displacement increases the association between raptor and insulin receptor substrate-1 (IRS-1), facilitating IRS-1 phosphorylation at serine 636/639. Finally, both S473 and T308 phosphorylation of Akt are reduced during decidualization, followed by a decrease in forkhead box O1 (FOXO1) phosphorylation and an increase in the mRNA levels of the decidualization markers prolactin (PRL) and insulin-like growth factor-binding protein-1 (IGFBP-1). Taken together, our findings reveal a critical role for mTOR in decidualization, involving the differential regulation of mTORC1 and mTORC2.

## Fertility: How cells lining the uterus prepare for pregnancy

A key signaling pathway plays a critical role in readying cells of the endometrium, the uterine lining, for embryo implantation and pregnancy. Mammalian target of rapamycin (mTOR) is an enzyme that links with other proteins to form two distinct protein complexes that regulate a variety of cellular processes. Researchers in Republic of Korea led by Joong-Soo Han from Hanyang University in Seoul, and Mee-Sup Yoon from Gachon University in Incheon found that levels of certain components within these two complexes decrease in the endometrium as cells change in size and shape in preparation for pregnancy. This process, they showed, also alters the pattern of phosphate groups on the mTOR complexes, ultimately leading to decreased overall activity of mTOR complex 2 but increased activity of mTOR complex 1. The findings could inform future development of fertility treatments.

## Introduction

Decidualization is the differentiation process of endometrial stromal (ES) cells, which determines the successful implantation of an embryo and the subsequent formation of a functional placenta^1^. This process involves the morphological transformation of fibroblast-like ES cells to enlarged decidual cells, which are biochemically and functionally distinct cells. Progesterone induces decidualization of human ES (hES) cells during the late phase of the menstrual cycle, and subsequent embryo implantation leads to and extends the persistent decidualization throughout the endometrium, forming the pregnancy decidua^2^. Decidualization is required to render the endometrium receptive to an incoming embryo and for pregnancy maintenance^3^. In vitro decidualization of human ES cells has been induced in the presence of hormones and growth factors, which mimic the in vivo decidual transformation. It has been shown that the activation of the intracellular cyclic monophosphate (cAMP) pathway as well as progesterone is essential for the decidualization of human ES cells^4^.

The mammalian target of rapamycin (mTOR) pathway regulates many cellular signaling and developmental processes in response to growth factors and nutrients^5^. mTOR forms two different complexes, known as mTOR complex 1 (mTORC1) and mTOR complex 2 (mTORC2)^5^. mTORC1 has three core components, namely, mTOR, raptor, and mLST8, which modulate translation and protein synthesis by phosphorylating ribosomal S6 kinase 1 (S6K1) and eukaryotic initiation factor 4E-binding protein1 (4EBP1)^6^. mTORC2 consists of mTOR, rictor, mLST8, and mSin1, and it regulates cell survival and cytoskeleton rearrangement by activating Akt, serum/glucocorticoid-regulated kinase, and protein kinase Cα^6^. In addition, mTOR activity is modulated by several mTOR-bound endogenous inhibitors, such as PRAS40 (mTORC1), XPLN (mTORC2), and DEPTOR (mTORC1 and mTORC2)^5,7^.

It has been reported that mTOR is a critical regulator of diverse differentiation processes, such as myogenesis^8,9^, adipogenesis^10,11^, T-cell differentiation^12^, and hepatic differentiation^13^, suggesting that it may also be involved in decidualization. The expression of mTOR increases in pregnant mice during early embryo implantation, mainly in the stromal cells^14^. In addition, the components of mTORC1 and mTORC2 are expressed in the uterus during pregnancy^15^. There is also evidence for the functional involvement of mTOR signaling during pregnancy. mTOR-associated cell signaling pathways mediate the interactions between the maternal uterus and peri-implantation conceptuses^15^. Treatment with rapamycin, a specific mTOR inhibitor, decreases the number of implantation sites^14^, suggesting the importance of mTOR for embryo implantation. Phosphorylation of mTOR at both serine 2448 and serine 2481 increases the placentation sites in mouse^16^, whereas phosphorylation of mTOR at serine 2481 (pS2481-mTOR) decreases during 8-bromoadenosine 3’,5’-cyclic monophosphate (8-Br-cAMP)-induced decidualization of human ES cells^17,18^. Arginine and leucine are sufficient to induce blastocyte activation, which is dependent on rapamycin-independent mTORC1 activity^19^. Moreover, mTORC1 has been shown to determine the timing of birth and fetal death in mice^20^. The interaction between p53 and sestrin activates 5’-AMP-activated protein kinase (AMPK), resulting in the inhibition of mTORC1 and preterm birth^21^. These results suggest that regulation of mTOR is crucial for maintaining pregnancy. Although these observations suggest a potential role of mTOR in implantation, the formation of decidua, and the maintenance of pregnancy, the underlying mechanisms regulating mTORC1 and mTORC2 activity have never been elucidated.

In the present study, we investigated the involvement of mTORC1 and mTORC2 in the differentiation of human ES cells. We show that mTORC1 and mTORC2 differentially modulate 8-Br-cAMP-induced decidualization of hES cells. We found that, during this differentiation process, mTORC1 is activated by DEPTOR displacement, resulting in dampened pT308-Akt expression and that concurrently, mSin1 dissociates from mTORC2, resulting in a reduction in pS473-Akt expression. The reduction in the phosphorylation of Akt leads to the subsequent activation of forkhead box O1 (FOXO1) and the expression of the decidualization markers, prolactin (PRL) and insulin-like growth factor-binding protein-1 (IGFBP1). These results provide evidence that mTOR regulation of the activity of the Akt-FOXO1 signaling pathway is essential for the process of decidualization in hES cells.

## Materials and methods

### Antibodies and other reagents

Antibodies were obtained from the following sources: FLAG M2 from Sigma-Aldrich (St. Louis, MO); DEPTOR from Novus; tubulin from Abcam (Cambridge, UK); raptor, rictor, and mSin1 from Bethyl laboratory (Montgomery, TX); mTOR from Santa Cruz Biotechnology (Santa Cruz, CA); and all other antibodies from Cell Signaling Technology (Danvers, MA). All secondary antibodies were from Jackson ImmunoResearch Laboratories Inc. (West Grove, PA). 1,2-Dioctanoyl-sn-glycero-3–PA was obtained from Avanti Polar Lipids (Alabaster, AL). All other reagents were from Sigma-Aldrich (St. Louis, MO).

### Isolation and culture of hES cells

hES cells were isolated from human endometrium, which was obtained by hysterectomy from 25 premenopausal women aged 40–45 years. The participants underwent surgery for nonendometrial abnormalities at Hanyang University Hospital between September 2008 and September 2014. Histological examination was conducted for each endometrial specimen. Isolation of hES cells was performed following a previously described procedure^22^. hES cells were grown in Dulbecco’s-modified Eagle’s medium (DMEM) containing 1 g/L glucose with 10% fetal bovine serum at 37 °C with 5% CO_2_. To induce in vitro decidualization, cells were plated on tissue culture plates, grown to 100% confluence, treated with differentiation medium (DMEM containing 0.5 mM 8-Br-cAMP) and given with fresh medium daily for 2 days.

### Lentivirus-mediated short hairpin (sh) RNA and transfection

DEPTOR shRNAs contained in the pLKO.1-puro vector were obtained from Sigma-Aldrich (MISSION shRNA). The clone IDs were as follows: DEPTOR-1, TRCN0000168212; DEPTOR-2, TRCN0000240949. The shRNAs for raptor and rictor were obtained from Addgene. Lentivirus packaging and testing were performed as previously described^9^. hES cells were transduced with lentiviruses in growth medium containing 8 μg/mL polybrene and 2 μg/mL puromycin for selection for 5 days, and they were plated in 12 well plates for differentiation. For transfection with DEPTOR, cells were transfected with Flag-DEPTOR^23^ using TransIT-LT1 (Mirus Bio LLC, Madison, US), following the manufacturer’s recommendations.

### Cell lysis, immunoprecipitation, and western blot analysis

hES cells were washed once with ice-cold phosphate buffered saline and were then lysed with lysis buffer (Cell Signaling Technology, Danvers, MA). The supernatant, after microcentrifugation at 13,000 × *g* for 10 min, was collected and then boiled in a sodium dodecyl sulfate (SDS) sample buffer for 5 min. Immunoprecipitation was performed with anti-rictor or anti-raptor antibody and then incubated with protein G agarose for 1 h at 4 °C. Lysis buffer containing 40 mM 4-(2-hydroxyethyl)-1-piperazineethanesulfonic acid (HEPES) (pH 7.4), 120 mM NaCl, 10 mM pyrophosphate, 50 mM NaF, 10 mM β-glycerophosphate, 2 mM EDTA, 1X Sigma protease inhibitor cocktail, and 0.3% 3-[(3-cholamidopropyl)dimethylammonio]-1-propanesulfonate (CHAPS) was used for immunoprecipitation. The beads were washed with lysis buffer three times and then boiled in SDS sample buffer for 5 min. Proteins were resolved on SDS-polyacrylamide gels and transferred onto polyvinylidene fluoride membranes (Millipore, Billerica, MA). Antibody incubations were performed following the manufacturer’s recommendations. Horseradish peroxidase-conjugated secondary antibodies were detected with Chemiluminescent HRP Substrate (Millipore, Billerica, MA). Images were developed using X-ray film.

### Quantitative real-time (RT)-PCR

Total RNA was extracted from either undifferentiated or differentiating hES cells using TRIzol reagent (Thermo Fisher Scientific, Waltham, MA). cDNA was synthesized from 1 μg RNA using TOPscript^TM^ RT DryMIX kit (dT18 plus) (Enzynomics, Daejeon, Korea). Real-time PCR analysis was performed with a CFX384 C1000 Thermal Cycler (Bio-Rad, Hercules, CA) using TOPreal^TM^ qPCR 2X PreMIX (SYBR Green with high ROX) (Enzynomics, Daejeon, Korea). Human glyceraldehyde 3-phosphate dehydrogenase (GAPDH) was used to normalize gene expression. A list of primer sequences is provided in Table 1.

**Table 1:** Primers used in this study

Gene	Sequence
*PRL F*	GGAGCAAGCCCAACAGATGAA
*PRL R*	GGCTCATTCCAGGATCGCAAT
*IGFBP1 F*	TTGGGACGCCATCAGTACCTA
*IGFBP1 R*	TTGGCTAAACTCTCTACGACTCT
*FOXO1 F*	GGATGTGCATTCTATGGTGT
*FOXO1 R*	TTTCGGGATTGCTTATCTCA﻿
*DEPTOR F*	CTCAGGCTGCACGAAGAAAAG
*DEPTOR R*	TTGCGACAAAACAGTTTGGGT
*GAPDH F*	GGAGCGAGATCCCTCCAAAAT
*GAPDH R*	GGCTGTTGTCATACTTCTCATGG

### Statistical analysis

All data are presented as the mean ± standard deviation (SD). Where necessary, statistical significance was determined by performing a one-sample *t*-test. *P*-values of < 0.05 were considered statistically significant.

## Results

### mTORC1 and mTORC2 differentially regulate 8-Br-cAMP-induced decidualization

**Fig. 1:**
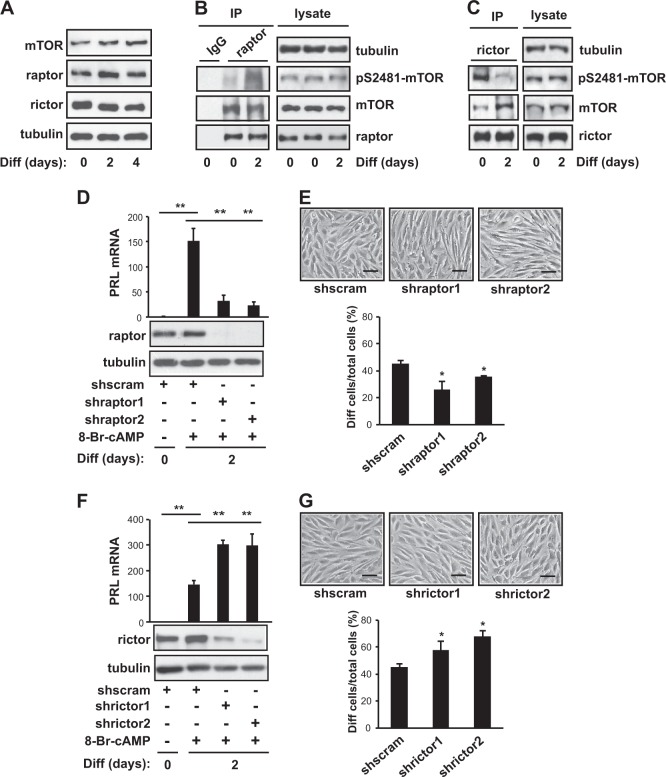
mTORC1 and mTORC2 differentially regulate 8-Br-cAMP-induced decidualization. **a** Human endometrial stromal (hES) cells were induced to differentiate for 4 days in the presence of 0.5 mM 8-Br-cAMP. On day 0, 2, or 4 of differentiation, the cells were lysed and subjected to western blotting. **b**, **c** On day 0 or 2 of differentiation, the cells were lysed, subjected to immunoprecipitation against raptor (**b**) or rictor (**c**) and were analyzed by western blotting. **d–g** hES cells were infected with lentiviruses expressing two different raptor shRNAs (**d**, **e**), rictor shRNAs (**f**, **g**), or scrambled shRNA. They were then selected by puromycin for 5 days and differentiated for 2 days in the presence of 0.5 mM 8-Br-cAMP. **d**, **f** The cells were lysed and analyzed by either quantitative RT-PCR or western blotting. Human GAPDH was used to normalize gene expression. **e**, **g** Cells were photographed under bright-field illumination. Round-shaped cells were scored as differentiated cells. Scale bar = 50 μm. **a–g** Data are representative of three to four independent experiments. **d**-**g** Data are expressed as the mean ± SD, with paired *t*-tests performed as indicated. *P < 0.05, **P < 0.01

To gain insight into the involvement of mTOR signaling in successful embryo implantation and pregnancy, we assessed mTOR signaling during in vitro decidualization, a process that is closely related to stromal differentiation in vivo^1^. Primary hES cells grown to 100% confluence were induced to differentiate using 0.5 mM 8-Br-cAMP. Typically, mRNA expression of the decidualization markers, i.e., PRL, IGFBP1, and FOXO1 (Supplementary Fig. 1A), and morphological changes (Supplementary Fig. 1B) were evident 2–3 days after induction. The protein levels of mTOR, raptor, and rictor, the main components of mTORC1 and mTORC2, remained unchanged during 8-Br-cAMP-induced decidualization (Fig. 1a). Next, to investigate any potential change in mTOR kinase activity, we examined mTOR phosphorylation on S2481, an autophosphorylation site that has been reported to monitor mTOR-specific catalytic activity^24^. To distinguish between pS2481-mTORC1 and pS2481-mTORC2, we isolated mTORC1 and mTORC2 by immunoprecipitation using anti-raptor and -rictor, respectively. S2481 phosphorylation of raptor-associated mTOR (mTORC1) increased during 8-Br-cAMP-induced decidualization (Fig. 1b). On the other hand, rictor-associated mTOR (mTORC2) had high basal levels of S2481 phosphorylation, which decreased drastically 2 days after the induction of differentiation (Fig. 1c).

Next, in order to investigate whether mTORC1 or mTORC2 is required for the differentiation of hES cells, we used two independent shRNAs targeting either raptor or rictor and a scramble hairpin sequence as a negative control. Interestingly, knockdown of raptor decreased the expression of PRL, a well-known marker of decidualization (Fig. 1d), and reduced the number of decidual cells with a round-shaped morphology (Fig. 1e). However, knockdown of rictor enhanced PRL expression (Fig. 1f) and the number of round-shaped decidual cells (Fig. 1g). These results suggested that an increase of mTORC1 activity or a decrease of mTORC2 activity is critical for decidualization.

### mTORC1 phosphorylates insulin receptor substrate-1 (IRS-1) at S636/639 during 8-Br-cAMP-induced decidualization

**Fig. 2:**
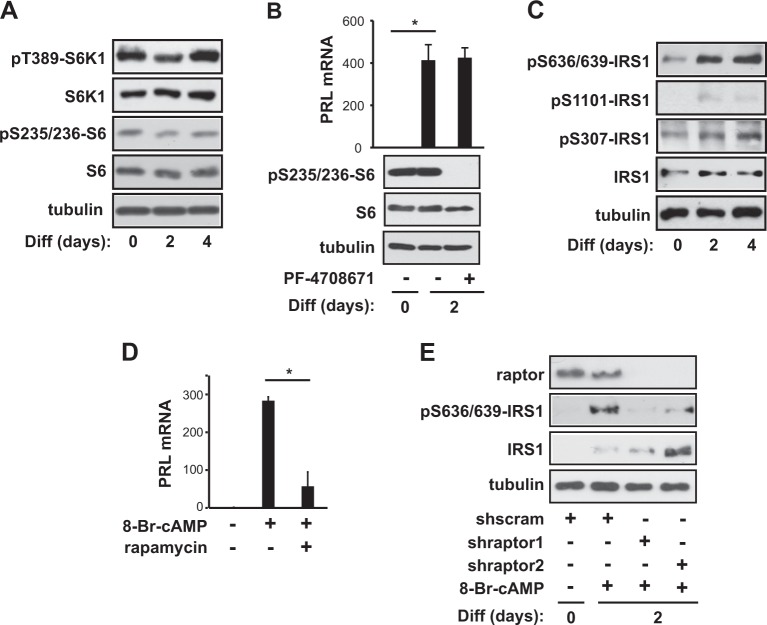
mTORC1 phosphorylates IRS-1 at S636/639 during 8-Br-cAMP-induced decidualization. **a** Human endometrial stromal (hES) cells were induced to differentiate for 4 days in the presence of 0.5 mM 8-Br-cAMP. On day 0, 2, or 4 of differentiation, the cells were lysed and subjected to western blotting. **b** Cells were differentiated with or without 15 μM PF-4708671 at the initiation of differentiation for 2 days. Cells were lysed and then analyzed by either quantitative RT-PCR or western blotting. **c** On day 0, 2, or 4 of differentiation, the cells were lysed and subjected to western blotting. **d** Cells were differentiated for 2 days with or without 100 nM rapamycin at the initiation of differentiation for 2 days. **e** hES cells were infected with lentiviruses expressing two different raptor shRNAs or scrambled shRNA; cells were then selected by puromycin for 5 days. After 2 days of differentiation, the cells were lysed and then analyzed by either quantitative RT-PCR or western blotting. Human GAPDH was used to normalize gene expression. All blots are representative of three to five independent experiments. All data are shown as the mean ± SD, with paired *t*-tests performed as indicated. *P < 0.05

Next, we evaluated the phosphorylation of T389-S6K1, a well-established downstream target of mTORC1, since mTORC1 autokinase activity increased during decidualization (Fig. 1b). Interestingly, S6K1 phosphorylation at T389 and subsequent S6 phosphorylation at S235/236 remained unchanged during 8-Br-cAMP-induced differentiation of hES cells (Fig. 2a). In addition, treatment with PF-4708671, an S6K1 inhibitor, did not affect the PRL mRNA expression level 2 days after the induction of differentiation, though the effectiveness of the S6K1 inhibitor was confirmed by the almost complete inhibition of S6 phosphorylation (Fig. 2b). These results indicated that S6K1 is dispensable for 8-Br-cAMP-induced decidualization and that mTORC1 might regulate decidualization via downstream targets other than S6K1. To identify such mTORC1-controlled downstream targets in decidualization, we examined the phosphorylation of IRS-1 at various serine residues (S307, S636/639, and S1101). mTORC1 is known to phosphorylate IRS-1 at serine residues, resulting in the degradation of IRS-1 or the reduction of IRS-1 signaling to phosphoinositide 3-kinase (PI3K)/Akt^25^. As shown in Fig. 2c, IRS-1 protein levels increased during cAMP-induced differentiation of hES cells, consistent with previous findings^26^. At the same time, IRS-1 phosphorylation at S636/639 obviously increased, whereas phosphorylation at both S307 and S1101 mildly increased, most likely as a reflection of the increase in the IRS-1 protein level. In addition, treatment with rapamycin severely reduced the mRNA level of PRL, a decidualization marker, during cAMP-induced decidualization (Fig. 2d). Consistent with mTORC1 phosphorylation of IRS-1 during cAMP-induced decidualization, the depletion of raptor reduced the S636/639 phosphorylation of IRS-1 during cAMP-induced decidualization and, concurrently, increased the IRS-1 protein level (Fig. 2e), leading to a reduction in the PRL mRNA level (Fig. 1d). These results suggested that mTORC1 enhances decidual transformation by increasing the S636/639 phosphorylation of IRS-1.

### DEPTOR displacement from mTORC1 increases the association between raptor and IRS-1

**Fig. 3:**
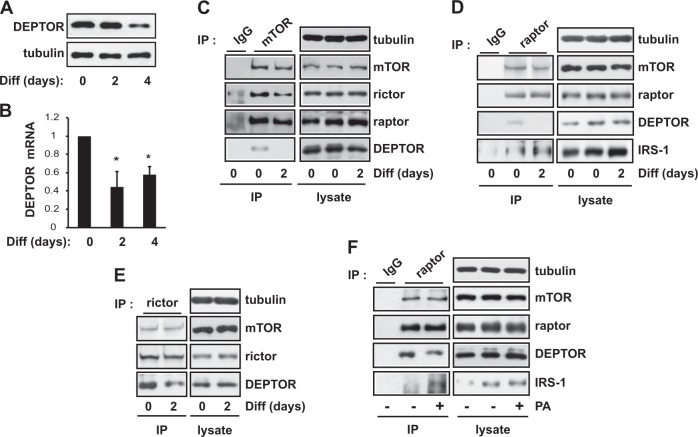
DEPTOR displacement from mTORC1 increases the association between raptor and IRS-1. **a**, **b** Human endometrial stromal (hES) cells were induced to differentiate for 4 days in the presence of 0.5 mM 8-Br-cAMP. On day 0, 2, or 4 of differentiation, the cells were lysed and subjected to western blotting (**a**) and quantitative RT-PCR (**b**) to analyze DEPTOR expression. Human GAPDH was used to normalize gene expression. **c** On day 0 or 2 of differentiation, hES cells were lysed, subjected to immunoprecipitation against mTOR, and analyzed by western blotting. **d**, **e** Cells were treated as in (**c**), lysed, subjected to immunoprecipitation against either raptor (**d**) or rictor (**e)**, and then analyzed by western blotting. **f** Cells were treated with 300 μM C8-PA for 30 min, lysed, subjected to immunoprecipitation against raptor, and analyzed by western blotting. All data are shown as the mean ± SD or are blots representative of three to five independent experiments. Student’s *t*-tests were performed to compare the indicated pairs of data. *P *<* 0.05

To elucidate how mTORC1 kinase activity increases, we analyzed the amount of DEPTOR, an endogenous dual inhibitor of mTORC1 and mTORC2, in mTOR complexes. As shown in Fig. 3a and b, the protein and mRNA levels of DEPTOR declined during 8-Br-cAMP-induced decidualization. Notably, the amount of DEPTOR associated with immunoprecipitated mTOR decreased after 2 days of decidualization (Fig. 3c). In mTORC1 complexes specifically isolated by immunoprecipitation with an antibody against raptor, the amount of DEPTOR was reduced after 2 days of differentiation (Fig. 3d), whereas the level of DEPTOR in mTORC2 isolated by immunoprecipitation with an antibody against rictor remained relatively unchanged (Fig. 3e). In addition, the amount of mTOR in mTORC1 in decidual cells was similar to that of undifferentiated cells (Fig. 3d). These results indicate that the increased mTORC1 kinase activity we observed during the differentiation of hES cells was a result of the displacement of DEPTOR from mTORC1.

Because raptor binding to the SAIN (Shc and IRS-1 NPXY binding) domain of IRS-1 is known to regulate phosphorylation of S636/639-IRS-1 by mTOR^27^, we investigated whether the interactions between raptor and IRS-1 increased during 8-Br-cAMP-induced decidualization. As shown in Fig. 3d, raptor directly interacts with IRS-1, as reported previously^27^, and their interaction was further increased 2 days after the induction of decidualization, coinciding with the displacement of DEPTOR from mTORC1. We propose that DEPTOR displacement from mTORC1 further facilitates the interaction between raptor and IRS-1 and, subsequently, the phosphorylation of IRS-1 at S636/639 (Fig. 2c).

It has been reported that phospholipase D1 (PLD1)-produced phosphatidic acid (PA) induces DEPTOR displacement^23^. PLD1-produced PA, which contains at least one unsaturated fatty acid chain, binds to the FRB domain of mTOR and displaces DEPTOR from mTORC1 to activate mTORC1 kinase activity^23^. Interestingly, the protein level and activity of PLD1 have been shown to increase during decidualization in previous studies^22,28^. Therefore, we tested whether PA enhances the interaction between raptor and IRS-1 while displacing DEPTOR from mTORC1. We chose a short-chain PA (C8-PA) in order to avoid the conversion of PA to lysophosphatidic acid^29^. When cells were treated with C8-PA, DEPTOR was displaced from mTORC1, and simultaneously, the interaction between raptor and IRS-1 increased (Fig. 3f). These results imply that PLD1-produced PA induces DEPTOR displacement, resulting in an enhanced interaction between raptor and IRS-1, which augments the phosphorylation of IRS-1 at S636/639.

### DEPTOR is a negative regulator of decidualization

**Fig. 4:**
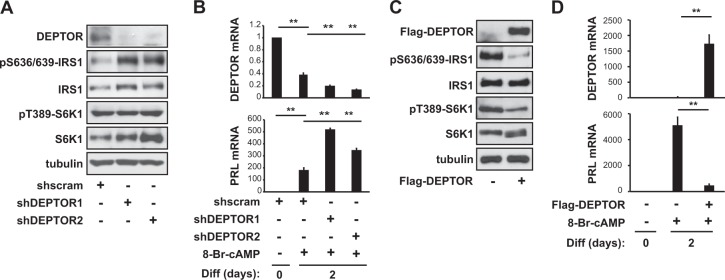
DEPTOR is a negative regulator of decidualization. **a**, **b** Human endometrial stromal (hES) cells were infected with lentiviruses expressing two different DEPTOR shRNAs or scrambled shRNA and were then selected by puromycin for 5 days. **a** Cells were lysed and then analyzed by western blotting. **b** After 2 days of differentiation, the cells were lysed and subjected to quantitative RT-PCR. **c** Cells were transfected with Flag-DEPTOR, lysed, and analyzed by western blotting. **d** Cells were treated as described in (**c**), differentiated for 2 days, and then subjected to quantitative RT-PCR. Human GAPDH was used to normalize gene expression. All data are shown as the mean ± SD or are blots representative of three to five independent experiments. Student’s *t*-tests were performed to compare the indicated pairs of data. **P *<* 0.01

Next, we investigated whether DEPTOR plays a role in regulating decidualization. Although DEPTOR is a dual inhibitor of mTORC1 and mTORC2, it primarily affects mTORC1 rather than mTORC2 activity^30^. However, the effect of DEPTOR on mTORC1 activity is mild, unlike the potent inhibition of mTORC1 due to raptor knockdown or rapamycin treatment^31^. We investigated the effect of DEPTOR knockdown by lentivirus-delivered shRNA, using two independent shRNAs for DEPTOR and a scramble hairpin sequence as a negative control. Knockdown of DEPTOR by two independent shRNAs increased pS636/639-IRS-1 without affecting either pT389-S6K1 (Fig. 4a) or pS473-Akt (data not shown), indicating that DEPTOR regulates mTORC1’s interactions with IRS-1 in this context. When hES cells transfected with DEPTOR shRNAs were induced to differentiate using 8-Br-cAMP, the mRNA levels of PRL (Fig. 4b) and IGFBP1 (Supplementary Fig. 2A) increased compared to control, alongside an enhancement of decidua-like morphology (Supplementary Fig. 2B). Conversely, overexpression of DEPTOR completely blocked pS636/639-IRS1 (Fig. 4c) as well as 8-Br-cAMP-induced decidualization, as indicated by the mRNA expression levels of PRL (Fig. 4d) and IGFBP1 (Supplementary Fig. 2C). These results imply that DEPTOR negatively regulates decidualization by inhibiting mTORC1 kinase activity on IRS-1 at S636/639.

### Akt inactivation during decidualization is accompanied by decreased pS256-FOXO1

**Fig. 5:**
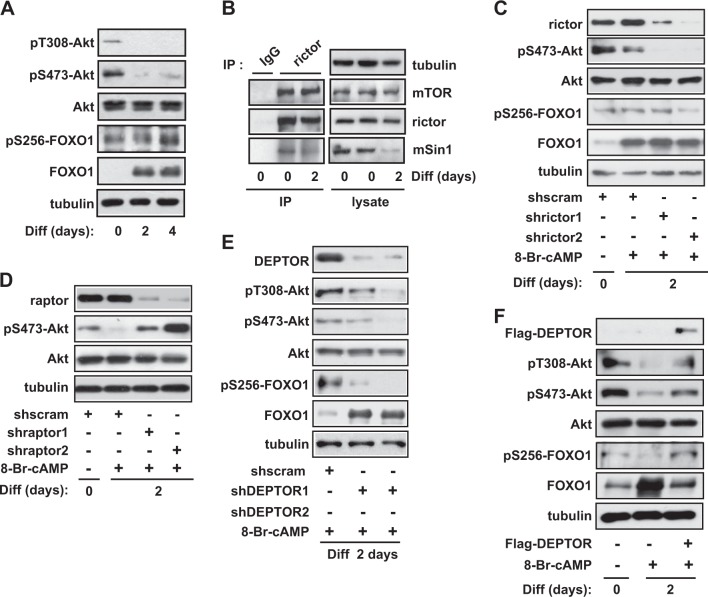
Differential regulation of mTORC1 and mTORC2 inactivates Akt, accompanied by decreased pS256-FOXO1 during 8-Br-cAMP-induced decidualization. **a** Human endometrial stromal (hES) cells were differentiated with 8-Br-cAMP for 4 days, lysed, and analyzed by western blotting. **b** On day 0 or 2 of 8-Br-cAMP-induced differentiation, hES cells were lysed, subjected to immunoprecipitation against rictor, and analyzed by western blotting. **c–e** Cells were infected with rictor shRNAs (**c**), raptor shRNAs (**d**), DEPTOR shRNAs (**e**), or scrambled shRNA; cells were then selected by puromycin for 5 days. Cells were differentiated in the presence of 0.5 mM 8-Br-cAMP, lysed, and then subjected to western blotting. **f** Cells were transfected with Flag-DEPTOR, differentiated with 0.5 mM 8-Br-cAMP for 2 days, lysed, and then subjected to western blotting. All blots are representative of three to five independent experiments

The active form of IRS-1 activates PI3K, producing phosphatidylinositol (3,4,5)-trisphosphate (PtdIns(3,4,5)*P*_3_), leading to 3-phosphoinositide-dependent protein kinase-1 (PDK1) activation and Akt phosphorylation at T308^32^. Phosphorylation of IRS-1 at its serine residues induces its degradation and blocks its activation^25^. During 8-Br-cAMP-induced decidualization, pT308-Akt drastically decreased (Fig. 5a), presumably due to the negative feedback loop of IRS-1 inhibition resulting from the phosphorylation of S636/639 by mTORC1. Akt phosphorylation at S473, an mTORC2 phosphorylation site, was also completely inhibited (Fig. 5a), which is consistent with the observed inactivation of mTORC2 kinase activity (Fig. 1c). Then, we analyzed the phosphorylation of S256-FOXO1, which is a target site of Akt^33^. FOXO1 is known as a marker of decidualization and functions as a critical transcription factor of other decidual marker genes during decidualization, including PRL and IGFBP1^34,35^. The phosphorylation of FOXO1 by Akt is known to reduce its transcriptional activity^36^. We observed that FOXO1 expression remarkably increased after the induction of decidualization (Fig. 5a), as shown in previous studies^34,37^, which is consistent with the increase in FOXO1 mRNA levels we observed (Supplementary Fig. 1A). Despite the increased expression of total FOXO1 protein, the pS256-FOXO1 level was not significantly increased but, rather, decreased during decidualization when the ratio of pS256-FOXO1/FOXO1 was examined (Supplementary Fig. 3A). These results suggested that Akt inactivation is correlated with the reduction of pS256-FOXO1 in decidual cells.

### Differential regulation of mTORC1 and mTORC2, which results in Akt inactivation and is required for decidualization

Since we observed a decrease in mTORC2 kinase activity (Fig. 1c) and in the phosphorylation of the mTORC2 substrate S473-Akt (Fig. 5a), without any change in mTOR or DEPTOR levels in mTORC2 during decidualization of hES cells (Fig. 3e), we analyzed the level of the mTORC2 component mSin1, a protein that is required for mTORC2 assembly and Akt phosphorylation^38^. The mSin1 protein level completely decreased 4 days after induction of differentiation (Supplementary Fig. 3B). When mTORC2 was isolated through immunoprecipitation with antibody against rictor, the level of mSin1 in mTORC2 decreased 2 days after 8-Br-cAMP-induced differentiation (Fig. 5b). To examine the role of mTORC2 activity in 8-Br-cAMP-induced decidualization, we downregulated rictor expression using two independent shRNAs combined with a lentiviral delivery system. As shown in Fig. 5c, the knockdown of rictor drastically reduced pS473-Akt and caused a decrease in pS256-FOXO1 levels greater than the decrease observed in control cells. This decreased phosphorylation was followed by an increase in PRL mRNA levels (Fig. 1f) and the enhancement of decidua-like morphology (Fig. 1g), suggesting that the decrease of Akt activity and pS256-FOXO1 levels by mTORC2 inactivation contributes to decidualization. In contrast, raptor knockdown enhanced pS473-Akt (Fig. 5d), resulting in a decrease in PRL mRNA levels (Fig. 1d) and decidua-like morphology (Fig. 1e), further indicating that the downregulation of Akt activity contributes to decidual events. Moreover, knockdown of DEPTOR further decreased the phosphorylation of T308-Akt, S473-Akt, and S256-FOXO1 and was followed by a significant increase in FOXO1 protein levels (Fig. 5e). However, the overexpression of DEPTOR augmented phosphorylation of Akt and S256-FOXO1 (Fig. 5f). These results suggest that the inactivation of Akt by differential regulation of mTORC1 and mTORC2 is essential for 8-Br-cAMP-induced decidualization by decreasing the pS256-FOXO1 level.

## Discussion

Decidualization is a multistage process that is tightly regulated by well-orchestrated signaling pathways. In the present study, we show that both mTORC1 activation and mTORC2 inactivation are involved in 8-Br-cAMP-induced decidualization. In this biological context, the regulation of mTOR activities decreased Akt activity and FOXO1 phosphorylation at S256, leading to differentiation in hES cells.

Although both mTORC1 and mTORC2 are often involved in the same biological contexts and processes, each complex contributes to regulation through distinct mechanisms. At the molecular level, mTORC1 differs from mTORC2 in its stability^39^, its localization^40^, and the effect of DEPTOR on its activity^23^. Distinct differences in the activity of the mTOR complexes are also evident at the physiological level. In oligodendrocyte differentiation, mTORC1 mediates the generation of mature oligodendrocytes by regulating the translation of myelin genes, while mTORC2 transcriptionally controls key genes required for oligodendrocyte differentiation^41^. In another context, a recent report showed that a positive feedback loop between sestrin2 and mTORC2 inhibits mTORC1 activity in glutamine-depleted lung cancer cells, preventing ATP depletion and maintaining the redox balance^42^. In addition, mTORC1 and mTORC2 have distinct roles in determining mesenchymal stem cell (MSC) lineage commitment; raptor knockout reduces the adipogenic differentiation potential and increases the osteogenic differentiation capacity, whereas rictor knockout has the opposite effect^43^. In line with these reports, the current study reveals that the assembly and activity of the mTORC1 and mTORC2 complexes have different contributions to the decrease in Akt activity during 8-Br-cAMP-induced decidualization.

cAMP cross-talk with several growth factor-induced pathways is expected^44^. More specifically, the relationship between the cAMP and mTOR signaling pathways has been the subject of many studies. It has long been understood that mTOR activity is dependent on cAMP-induced mitogenesis^45^. cAMP-dependent mTORC1 activation correlates with cAMP-induced activation of Akt in rat ovarian granulosa cells^46^, whereas cAMP-mediated inhibition of S6K1 and 4EBP1 is correlated with the blockage of the c-Raf/extracellular signal-regulated kinases (ERK) cascade in the thyroid papillary carcinoma cell line TPC-1^47^. The inhibition of mTOR signaling has also been observed in HEK293 cells; cAMP decreased S2481 phosphorylation of both mTORC1 and mTORC2 in the presence of growth factors, accompanied by the dissociation of both raptor and rictor from mTOR^48^. However, in the current study, cAMP-induced activation of mTORC1 was not correlated with Akt activity (T308 and S473) or the dissociation of either mTOR-raptor or mTOR-rictor. These results suggested that mTOR activity in hES cells is regulated by cAMP in a distinct manner compared to other biological contexts.

Notably, the present study demonstrates that the levels of bound DEPTOR and mSin1 modulate the activity of mTORC1 and mTORC2, respectively, during decidualization of hES cells. DEPTOR has already been found to regulate mTORC1 activity during the differentiation of several other cell types^31,49–51^. DEPTOR also functions to maintain the pluripotency of embryonic stem cells^49^. In addition, an increase in DEPTOR levels results in the inhibition of mTORC1 activity and in the negative feedback inhibition of IRS-1 during adipogenic and osteogenic differentiation of human adipose-derived MSCs^31,50^. We observed that, in the decidualization context, mTORC1 preferentially regulates IRS-1 phosphorylation at S636/639, probably due to the increased interactions between IRS-1 and raptor during decidualization (Fig. 3c). PA, which is produced by PLD1 during 8-Br-cAMP-induced decidualization^22,52^, induces DEPTOR displacement and facilitates the recruitment of IRS-1 to raptor during decidualization (Fig. 3f). Therefore, this finding demonstrates the mechanism through which PA is directly involved in IRS-1 regulation. To our knowledge, the present study constitutes the first report of the role of mSin1 in differentiation. mSin1 is indispensable for the assembly of mTORC2 and for its kinase activity towards Akt^53^. How mSin1 binding and mTORC2 activity are downregulated during decidualization is unclear. This downregulation may simply be a result of the decreased mSin1 protein levels we observed in decidual cells (Supplementary Fig. 3B). Recently, Yang et al.^54^ suggested the phosphorylation of mSin1 by PDK1-activated Akt as a mechanism that enhances mTORC2 kinase activity towards S473-Akt during growth factor stimulation. However, the findings of Yang et al. indicate that mSin1 binding to mTORC2 is not affected by pT86-mSin1, and therefore the lack of mSin1 binding we observed is unlikely to be a direct effect of the decreased Akt activity that accompanies decidualization. Therefore, it appears that both mSin1 binding and T86-mSin1 phosphorylation regulate mTORC2 kinase activity. Thus, we propose that the differential regulation of both mTOR complexes, the activation of mTORC1 and the inactivation of mTORC2, achieved by the modulation of the signaling complex composition, is critical for decidualization.

We found that the inactivation of Akt by mTORC1 activation and mTORC2 inactivation is indispensable for successful decidualization. The increased phosphorylation of S473-Akt dampened cAMP-induced decidualization in the absence of raptor (Figs. 1d, e and  5d). Consistent with this result, knockdown of rictor in differentiating hES cells further decreased pS473-Akt levels, resulting in enhanced expression of PRL (Figs. 1f and  5c). Supporting our observations, treatment with Akti-1/2, an Akt inhibitor, has been shown to augment decidua-like morphology and PRL and IGFBP1 expression^35^. Downregulation of Akt activity has been previously shown to be critically important for the establishment decidualization in vivo and in vitro in mice and rats^55,56^. Both the expression level and activity of Akt are reduced during the decidualization of hES cells, leading to decreased cell motility^57^. PP2A binds to and dephosphorylates Akt during cAMP- or PA-induced decidualization^35^. In line with these observations, the pAkt level has been shown to decline in the mid-secretory phase^58^. Downregulation of Akt during decidualization reduces pS256-FOXO1, which leads to enhanced FOXO1 transcriptional activity (Fig. 5a and Supplementary Fig. 3A), which is known to be a major regulator of progesterone-dependent differentiation of human endometrium^34^. We observed that FOXO1 is significantly induced upon decidualization (Supplementary Fig. 1A and Fig. 5a), consistent with previous findings^37,59^. FOXO1 knockdown has been shown to perturb the expression of genes that are involved in the regulation of the cell cycle, in differentiating hES cells through genome-wide expression profiling^34,60^. Furthermore, FOXO1 is crucial for the induction of decidual marker genes, such as PRL, IGFBP1, WNT4, and LEFTY2^60^. In hES cells, FOXO1a also binds to either HoxA-10 or HoxA-11, which regulate either IGFBP1 or PRL expression, respectively, suggesting that core transcription factors, including FOXO1a, physically and functionally interact to control the expression of decidual genes^61^. Further examination of the role of mTOR, especially in the context of inhibition of Akt and activation of FOXO1a, may deepen our understanding of stromal cell differentiation, thereby informing therapeutic strategies for implantation- and decidualization-related problems.

## Electronic supplementary material

SFigure 1

SFigure 2

SFigure 3

Supplementary Figure Legends
