# Supplementary material for: Differential regulation of mTORC1 and mTORC2 is critical for 8-Br-cAMP-induced decidualization
Source: Exp Mol Med. 2018 Oct 30;50(10):1–11. doi: 10.1038/s12276-018-0165-3 (PMC6206090; doi:10.1038/s12276-018-0165-3)
Supplement: Supplementary file 4 — Supplementary Figure Legends [file 12276_2018_165_MOESM4_ESM.docx]

**Supplementary Figure Legends**

**SFigure 1. The expression of decidual markers and morphological changes upon 8-Br-cAMP-induced decidualization (related to Fig. 1)**

(A) Human endometrial stromal (hES) cells were induced to differentiate for 4 days in the presence of 0.5 mM 8-Br-cAMP. On day 0, 2, or 4 of differentiation, the cells were lysed and subjected to quantitative RT-PCR. Data are expressed as the mean ± SD, with paired *t*-tests performed as indicated. **P* <0.05. (B) Cells were treated as (A) and then photographed under bright-field illumination. Scale bar = 50 μm.

**SFigure 2. DEPTOR negatively regulates decidualization (related to Fig. 4)**

(A, B) Human endometrial stromal (hES) cells were infected with lentiviruses expressing two different DEPTOR shRNAs or scrambled shRNA; cells were then selected by puromycin for 5 days. After 2 days of differentiation, cells were lysed, analyzed by quantitative RT-PCR (A), and photographed (B). The percentage of differentiated cells was quantified for experiments shown in SFigure 2B, as described in Figure 1. Scale bar = 50 μm. (C) Cells were transfected with Flag-DEPTOR, differentiated for 2 days, and then analyzed by quantitative RT-PCR. Data are expressed as the mean ± SD, with paired *t*-tests performed as indicated. **P* <0.05 ***P* <0.01.

**SFigure 3. Both pS256-FOXO1/FOXO1 and mSin1 protein levels decrease in decidual cells (related to Fig. 5)**

(A) Human endometrial stromal (hES) cells were induced to differentiate for 4 days in the presence of 0.5 mM 8-Br-cAMP. On day 0, 2, or 4 of differentiation, the cells were lysed and subjected to western blotting. Western blot band intensities were analyzed using ImageJ software (National Institutes of Health, Bethesda, MD), and the relative ratio of pS256-FOXO1 and FOXO1 was calculated. (B) Cells were treated as in (A) and were subjected to western blotting for mSin1. All data are shown as the mean ± SD or are blots representative of three to five independent experiments. Student’s *t*-tests were performed to compare the indicated pairs of data. **P* <0.05.
